# Reconfiguring global primary care evidence: The essential role of regional journals

**DOI:** 10.4102/safp.v68i2.6297

**Published:** 2026-03-18

**Authors:** Shailendra Prasad, Klaus B. von Pressentin, Bassim Birkland, Ramakrishna Prasad, Esther M. Johnston, Diego Garcia-Huidobro

**Affiliations:** 1 Department of Family Medicine and Community Health, Medical School, University of Minnesota, Minneapolis, United States; 2 The Global Engagement Network for Primary Health Care, Minneapolis, United States; 3 Center for Global Health and Social Responsibility, University of Minnesota, Minneapolis, United States; 4 Division of Family Medicine, Department of Family, Community and Emergency Care, University of Cape Town, Cape Town, South Africa; 5 Department of Family Medicine, University of Zambia, Lusaka, Zambia; 6 Seed Global Health Zambia, Lusaka, Zambia; 7 National Centre for Primary Care Research and Policy, Academy of Family Physicians of India, Bengaluru, India; 8 Department of Family Medicine, School of Medicine, Pontificiia Universidad Catolica de Chile, Santiago, Chile

**Keywords:** primary care research, regional journals, epistemic justice, health systems, global primary care

## Abstract

Primary care research (PCR) strengthens health systems and informs policy, but is dominated by high-income country (HIC) institutions and authors creating epistemic injustice by marginalising local knowledge from low- and middle-income countries (LMICs) and reinforcing structural inequities. Regional journals are vital for providing accessible, relevant evidence by lowering publishing barriers, supporting emerging researchers, enabling multilingual dissemination, and creating practice-focused knowledge for local health systems. Strengthening regional publishing is essential for a diverse, equitable global evidence base that improves the relevance, impact, and fairness of global PCR.

**Contribution:**

This article advances primary care scholarship by invoking epistemic justice and emphasising regional journals as vital for equitable knowledge. It references journals such as *South African Family Practice*, which celebrates 45 years of local research and mentorship. It links representation, research, and publishing, critiques citation metrics, and offers strategies to improve regional journals and promote justice in global primary care evidence.

## Introduction

Primary care research (PCR) plays a central role in shaping global health policy and advocacy, strengthening health systems, and informing front-line clinical practice. Yet the global primary care evidence base remains skewed towards institutions, authors and funding priorities in high-income countries (HICs).^1^ This imbalance raises essential questions about who generates authoritative knowledge, whose experiences are marginalised, and how global health scholarship perpetuates long-standing inequities. Scholars have increasingly described these patterns as forms of epistemic injustice – the systematic devaluation or exclusion of knowledge generated in low- and middle-income countries (LMICs) and the Global South more broadly.^2,3,4^

Regional journals have emerged as crucial platforms that challenge this global imbalance. They provide spaces where locally relevant, contextually grounded PCR can thrive. This opinion piece synthesises emerging scholarship on epistemic justice in global health and argues that regional journals should be strengthened as central actors in reshaping the landscape of primary care knowledge globally.

We suggest four themes to strengthen global PCR: (1) the need for more primary care publications globally; (2) epistemic justice and representation in publications; (3) the role of regional journals in promoting representation and locally relevant evidence; and (4) next steps for strengthening equity in global primary care knowledge production.

## The need for more primary care publications globally

Primary Health Care (PHC) is increasingly recognised as the most relevant, effective, and equitable foundation for health systems worldwide. Yet PHC research output remains disproportionately low relative to its contribution to population health.^1,5,6,7^ While high-income settings have robust publication infrastructures, researchers in LMICs often face barriers such as limited research funding, paucity of opportunities to develop primary care researchers through postgraduate pipelines, insufficient mentorship, a lack of protected time, constrained institutional research capacity, language barriers, and article processing charges that restrict access to high impact journals.^8,9^

The consequences of this imbalance are profound. The generated evidence for PHC guidelines, interventions, and implementation strategies may not translate effectively into most health systems.^10,11^ There is also the concern of a great deal of waste and redundancy in the evidence ecosystem.^12,13^ This undermines the global relevance of primary care literature and risks promoting interventions that are unfeasible, unaffordable, uninformed by evidence or misaligned with local needs and cultural values. Improved representation in primary care publications ensures that different *front-line realities – such as health worker shortages, rural service delivery, pluralistic health systems, and social determinants of health – shape global discourse*, rather than being retrofitted into frameworks derived from high-income nations.

Increasing PHC research output also strengthens local academic cultures. Publications support faculty promotion, guide curriculum development, and shape policy discussions at national and regional levels.^7^ Moreover, they supply the evidence needed to scale contextually appropriate innovations. Without more inclusive publication pathways, the global primary care literature will continue to perpetuate a narrow or distorted picture of what effective or equitable PHC looks like.

## Epistemic justice and representation in publications: Going beyond indices

The global health community increasingly recognises that inequities in knowledge production mirror broader colonial and structural power dynamics.^14,15^ Epistemic injustice manifests in multiple forms: underrepresentation of LMIC authors in high-impact journals, dominance of Western methodologies and English-language publishing, extractive authorship practices in global health collaborations, and privileging of biomedical over social, community-driven, or indigenous knowledge.^16,17,18,19^ These patterns reinforce what has been called *epistemic debt*: an unfair system in which LMIC contexts supply data and experiences, but not recognised expertise.^20^

The overreliance on metrics such as the h-index or journal impact factor deepens these inequities. Citation indices inherently reward established networks, English-language literature, and scholars situated in well-resourced institutions.^21^ Some of these indices may not capture the real-world impact of PHC research: improvements in service delivery, partnerships with communities, changes in clinical workflow or contributions to policy.^22^

For primary care – where research is often relational, community-embedded, and practice-oriented – these conventional metrics fail to reflect the true value of scholarship.^23^ However, the advent of the CRISP checklist could bolster the relevance of PHC research.^24^ Implementation research, participatory action research, indigenous knowledge systems, and practice-based evidence may have significant local impact, yet remain marginalised in global journals because of a perceived lack of novelty, methodological orthodoxy or limited generalisability.

Shifting the global research ecosystem towards epistemic justice requires valuing:

- *Contextual expertise* along with methodological expertise
- *Community-based participatory research*.
- *Local significance and implementation experiences* and global citation counts.
- Multilingual dissemination and publishing pathways that do not make English proficiency a prerequisite.
- Open-access and fee structures that do not exclude LMIC researchers from publishing or reading their own evidence.
- Editorial and peer-review boards that reflect geographic, linguistic, and epistemic diversity.
- *Equitable authorship structures* that reflect meaningful contributions.^25,26^
- *Diverse epistemologies*, including social sciences and indigenous knowledge.

Only then can PCR authentically reflect the lived realities of communities worldwide.

## The role of regional journals in advancing equity

Regional journals occupy a pivotal space in democratising global health knowledge. They counterbalance the dominance of HIC journals by elevating scholarship rooted in local experiences, health systems, and priorities.^13,27^ Several unique contributions distinguish regional journals from their global counterparts.

### A platform for contextually grounded evidence

Regional journals publish research that directly addresses the priorities of local health systems: rural care delivery, human resources for health, task-shifting, early pilots, hyperlocal adoption of innovations, integration of traditional medicine, district health management, community-based services, and social determinants of health.^28^ These topics are often sidelined by high-impact journals that prioritise generalisable methodological innovation over contextual relevance.

### Reduced barriers to access

Many regional journals offer:

- Reduced or waived publication fees.^29^
- Editorial support for early-career researchers.
- Encouragement and support to local practitioners.
- Flexibility in diverse methodologies.
- Openness to implementation and quality-improvement studies.
- multilingual abstracts or translation support.^30,31^

These features lower the threshold for participation, particularly for junior and mid-career researchers and practitioners, and for students, who may otherwise be excluded from global publication opportunities.

### A training ground for emerging scholars

Regional editorial boards provide mentorship in writing, reviewing, and scientific communication – skills central to developing national research capacity, along with editorial fellowships for new researchers and students.^32,33^ They also cultivate regional networks that link clinicians, researchers, and policymakers, enabling cross-country collaborations.

### Strengthening local policy and practice

Because regional journals are closely connected to local professional communities, their publications have high translational value. Ministry of Health policymakers, district managers, and front-line clinicians often utilise these journals for evidence directly applicable to their settings.^34^ This direct connection to practice is essential for advancing universal health coverage, improving PHC quality, and enabling rapid dissemination of innovations.

### Enabling knowledge exchange

Regional journals promote horizontal learning not just locally, but also among communities from across the world with similar health system structures, disease burdens or experiences of inequity in power and resources, who can learn from each other more effectively than through literature drawn from the contexts of more traditionally higher-resourced academic institutions and settings from which ‘high impact literature’ usually emerges.^35^

### Challenging epistemic hierarchies

By validating diverse epistemologies – including qualitative, ethnographic, participatory, and indigenous approaches – regional journals bring in varied, and relevant, knowledge production. This serves as a counterbalance to the dominance of narrow methodologies that can take the shape of hegemonies.^36^ They create a more pluralistic, just, and contextually relevant global evidence base.

## Next steps for strengthening equity in global primary care knowledge production

Advancing epistemic justice in global primary care scholarship requires coordinated action across institutions, journals, funders, and researchers.

### Reform academic incentives

Promotion and funding organisations must recognise diverse outputs beyond high-impact publications: policy contributions, community engagement, teaching, implementation studies, and international collaborations. This shift is critical for valuing the kinds of research that matter most to PHC. This would include deemphasising excessive focus on publication numbers and first and last authorship positions on articles.

### Invest in regional publishing infrastructures

Funders, usually professional organisations that sponsor regional journals, should support:

- Editorial capacity building.
- Digital publishing platforms.
- Wide indexing of regional journals.
- Open-access models without prohibitive author fees (e.g., institutional subscription or fee waiver criteria for locally produced knowledge that promotes diversity).^6^

A stronger regional publishing ecosystem will naturally expand LMIC representation in the global literature.

### Promote equitable authorship practices

Besides satisfying the International Committee of Medical Journal Editors (ICJME) criteria for publications, research collaborations must follow principles such as the Brocher Declaration and the Global Health Decolonization Movement in Africa (GHDMIA).^37,38^ These emphasise the importance of ethical engagement models and equity in participation in the research. Transparent credit allocation, early authorship discussions, and recognition of contextual expertise are essential to ensure equity in participation in research.

### Support national and regional research networks

Primary care research networks (PCRNs) facilitate multisite studies, nurture early-career researchers, and produce practice-relevant evidence.^39^ Other networks such as PRIMAFAMED, WONCA global and regional networks are important avenues to nurture research questions and develop PCR.^40^ Strengthening these networks will build a sustainable research culture across PHC settings.

### Facilitate multilingual dissemination

To democratise global health knowledge, journals should adopt multilingual abstracts, translation tools, and community-facing summaries. This is especially important for primary care literature intended for diverse audiences. Embedding artificial intelligence in journals can easily support this strategy.

### Align publication and research agendas with local priorities

Publication and research agendas must be set collaboratively with clinicians, communities, and policymakers to ensure relevance and direct application. Only then will published PCR meaningfully address the challenges faced by the populations it seeks to serve. Journals can integrate clinician-, student-, patient-, and community representatives into their editorial advisory boards to ensure that their voices are heard.

## Conclusion

Primary care research is indispensable for strengthening health systems, informing policy and advocacy, and achieving health equity. Yet, global primary care scholarship continues to suffer from epistemic injustice, with disproportionate representation from higher-income and higher-resourced settings and under-recognition of expertise from lower-resourced and marginalised communities.

Regional journals offer a powerful mechanism to correct this imbalance by elevating contextually grounded, practice-relevant evidence produced by local researchers. To build a more just and representative global evidence base, the academic community and funding agencies must move beyond narrow citation and publication metrics, invest in regional publishing ecosystems, and recognise the inherent value of diverse epistemologies. Strengthening regional journals is not a peripheral strategy; it is a central mechanism for generating evidence that is usable, contextually relevant, and transferable to the health systems where it is most needed.

The *South African Family Practice* journal, while officially representing the South African Academy of Family Physicians, has expanded its scope to include PCR from the Southern African region, aligning with the journal’s evolving editorial policy. This broader focus supports a global health perspective by sharing emerging evidence from neighbouring countries. The journal’s publisher supports the editorial team by tracking submissions regionally to bolster contextually relevant primary care evidence. Celebrating 45 years, this milestone emphasises the importance and potential of regional journals such as the *South African Family Practice* in shaping global primary care knowledge, serving as a platform for research, mentorship, and promoting equity in family medicine scholarship. It underscores the necessity of regional publishing to build a more inclusive global evidence base.
